# Supplementary material for: Demography and Natural Selection Have Shaped Genetic Variation in the Widely Distributed Conifer Norway Spruce (Picea abies)
Source: Genome Biol Evol. 2020 Jan 20;12(2):3803–17. doi: 10.1093/gbe/evaa005 (PMC7046165; doi:10.1093/gbe/evaa005)
Supplement: evaa005_Supplementary_Data [file evaa005_supplementary_data.zip › GBErevision-XiWang-diversity-Supplementaryfile-GBE-12-01-2019.docx]

# Supplemental materials

**Demography and natural selection have shaped genetic variation in the widely distributed conifer Norway Spruce (*Picea abies*)**

# Xi Wang^1,2^, Carolina Bernhardsson^1,2¶^, Pär K. Ingvarsson^2^*

^1^Umeå Plant Science Centre, Department of Ecology and Environmental Science, Umeå University, Umeå, Sweden

^2^Linnean Centre for Plant Biology, Department of Plant Biology, Swedish University of Agricultural Sciences, Uppsala, Sweden

^¶^Present address: Department of Organismal Biology, Uppsala University, Uppsala, Sweden

*Author for Correspondence: Pär K. Ingvarsson, Linnean Centre for Plant Biology, Department of Plant Biology, Uppsala BioCenter, Swedish University of Agricultural Science, Uppsala, Sweden, +46-18673230, [par.ingvarsson@slu.se](mailto:par.ingvarsson@slu.se)

Table S2. Summary statistics of genomic subsets with each included 35 individuals. Genomic length (mega bases) is the length of combined scaffolds in each subset; Average scaffold length (kilo bases) is the average length of each scaffold included in the subset; Unfiltered recodes (in millions) and unfiltered SNPs (in millions) are number of recodes and number of SNPs present in the unfiltered raw VCF files, respectively; Hard filtered SNPs (in millions) are number of SNPs retained after performing hard filtering criteria, and hard filtered scaffolds (%) are percentage of the scaffolds that contained only filtered SNPs in each subset.

| Subset | Genomic  Length  (Mb) | Average  Scaffold length (KB) | Unfiltered  Recodes  (Million) | Unfiltered  SNPs  (Million) | Hard filtered  SNPs  (Million) | Hard filtered  Scaffolds  (%) |
| --- | --- | --- | --- | --- | --- | --- |
| 1 | 2,654.8 | 26.6 | 230.7 | 217.7 | 114.1 | 96.1 |
| 2 | 1,657.9 | 16.6 | 113.2 | 107.8 | 44.1 | 80.1 |
| 3 | 451.9 | 4.5 | 40.6 | 38.4 | 15.1 | 77.6 |
| 4 | 394.3 | 3.9 | 35.4 | 33.4 | 12.9 | 76.2 |
| 5 | 481.1 | 4.8 | 43.9 | 41.4 | 16.4 | 79.4 |
| 6 | 243.3 | 2.4 | 21.7 | 20.5 | 7.6 | 70.4 |
| 7 | 245.3 | 2.5 | 22.0 | 20.7 | 7.7 | 70.6 |
| 8 | 257.8 | 2.6 | 23.2 | 21.9 | 8.4 | 71.9 |
| 9 | 329.2 | 3.3 | 27.5 | 25.8 | 11.3 | 64.7 |
| 10 | 256.4 | 2.8 | 7.8 | 7.6 | 0.5 | 16.5 |
| 11 | 241.0 | 2.4 | 13.0 | 12.6 | 1.5 | 33.8 |
| 12 | 159.1 | 1.6 | 13.3 | 12.6 | 3.9 | 55.5 |
| 13 | 179.2 | 1.8 | 15.0 | 14.2 | 4.5 | 56.6 |
| 14 | 196.9 | 2.0 | 16.5 | 15.7 | 5.3 | 57.9 |
| 15 | 213.0 | 2.1 | 17.9 | 17.0 | 6.0 | 58.5 |
| 16 | 229.6 | 2.3 | 19.2 | 18.2 | 6.6 | 59.7 |
| 17 | 237.6 | 2.4 | 17.6 | 16.8 | 5.2 | 59.3 |
| 18 | 262.2 | 2.6 | 20.1 | 19.2 | 6.1 | 66.3 |
| 19 | 331.3 | 3.3 | 25.7 | 24.4 | 8.4 | 66.1 |
| 20 | 433.6 | 6.2 | 25.3 | 23.6 | 8.2 | 39.5 |
| Total | 9,455.5 | 4.8 | 749.6 | 709.5 | 293.9 | 63.2 |

Table S3. Relative likelihood of the different demographic models shown in Figure S3.

| Model | Max  (log_10_(Lhood_i_))^a^ | No. of parameters (d) | AIC_i_^b^ | ∆_i_ ^b^ | Model normalized relative likelihood  (w_i_ )^b^ |
| --- | --- | --- | --- | --- | --- |
| No-Bot | -433738283 | 14 | 1997798560 | 1755486 | ~0 |
| Pop0-Bot | -433378378 | 22 | 1996140853 | 97779 | ~0 |
| Pop1-Bot | -433379115 | 22 | 1996144248 | 101174 | ~0 |
| Pop2-Bot | -433477940 | 22 | 1996599436 | 556362 | ~0 |
| Pop01-Bot | -433373421 | 28 | 1996118033 | 74959 | ~0 |
| Pop02-Bot | -433413182 | 28 | 1996301172 | 258098 | ~0 |
| Pop12-Bot | -433376659 | 26 | 1996132943 | 89869 | ~0 |
| Pop012-Bot^c^ | -433357145 | 32 | 1996043074 | 0 | 1 |

^a^ Based on the best likelihood among the 50 independent runs for each model.

^b^ The calculation of AIC_i_, ∆_i_ and w_i_ are according to the methods shown in Excoffier et al. (2013).

^c^ The best-fitting model was chosen based on ‘Model normalized relative likelihood (w_i_ )’ ≈ 1.

Table S4. Inferred parameters of demographic history in *P. abies* under best model-Pop012-Bot.

| Parameters | | | Point estimation | 95% CI^d^ | |
| --- | --- | --- | --- | --- | --- |
|  |  |  |  | Lower bound | Upper bound |
| Effective population  sizes^a^ | Ancestor | NANCTHR | 194773 | 76515 | 457249 |
|  |  | NANCSHR^e^ | 27223 | 2216 | 24608 |
|  |  | NANCTWO | 20211 | 616 | 327809 |
|  | Central-Europe | NPOPANC0^e^ | 60478 | 4325 | 47225 |
|  |  | NPOPBOT0 | 3393 | 209 | 5011 |
|  |  | NPOP0 | 311241 | 78122 | 424731 |
|  | Finland | NPOPANC1 | 92473 | 4041 | 453545 |
|  |  | NPOPBOT1 | 14992 | 179 | 41190 |
|  |  | NPOP1 | 1633 | 1036 | 376945 |
|  | Sweden-Norway | NPOPANC2 | 227495 | 11658 | 496858 |
|  |  | NPOPBOT2 | 17178 | 1429 | 44282 |
|  |  | NPOP2 | 4428 | 2482 | 6489 |
| Bottleneck  times^b^ | | TBOT012 | 28463 | 1650 | 103125 |
|  |  | TENDBOT12 | 30181 | 23788 | 39806 |
|  |  | TENDBOT0 | 101338 | 31144 | 109656 |
|  |  | TSHREND | 45632194 | 12720263 | 62706394 |
| Divergence  times^c^ | | TDIV1 | 32656 | 28669 | 44206 |
|  |  | TDIV2 | 114400 | 111238 | 119831 |

^a^ Effective population sizes for: the ancestor of three populations (NANCTHR), the ancestor of three populations experiencing bottleneck (NANCSHR), the ancestor of Finland and Sweden-Norway populations (NANCTWO), each population before bottleneck (NPOPANC), population experiencing bottleneck (NPOPBOT), and currently population (NPOP).

^b^ Time when bottleneck finished for three populations (TBOT012); time when bottleneck started for Finland and Sweden-Norway populations (TENDBOT12); time when bottleneck started for Central-Europe population (TENDBOT0); time when bottleneck started for ancestor of three populations (TSHREND).

^c^ Divergence time between Finland and Sweden-Norway populations (TDIV1); divergence time between Central-Europe population and the ancestor of Finland and Sweden-Norway populations (TDIV2).

^d^ Parametric bootstrap estimates obtained by parameter estimation from 100 datasets simulated according to the overall maximum composite likelihood estimates shown in point estimation columns. Estimations were obtained from 100,000 simulations per likelihood.

^e^ Two parameters were very ancient effective population sizes, so that point estimations were not inferred accurately and thus out of corresponding 95% CI ranges.

Figure S1. Geographic distribution of whole-genome re-sequenced individuals. Individuals of *P.abies* from Norway (yellow), Sweden (green), Finland (blue), Poland (orange), Belarus (red), and Romania (pink) are shown in circles with different colors. Individual Pab001 of *P. obovata* located in Yakutsk, Russia is shown by asterisk in the bottom right figure. Dark blue color represents the natural distribution of *P. abies* (EUFORGEN 2009,www.euforgen.org).


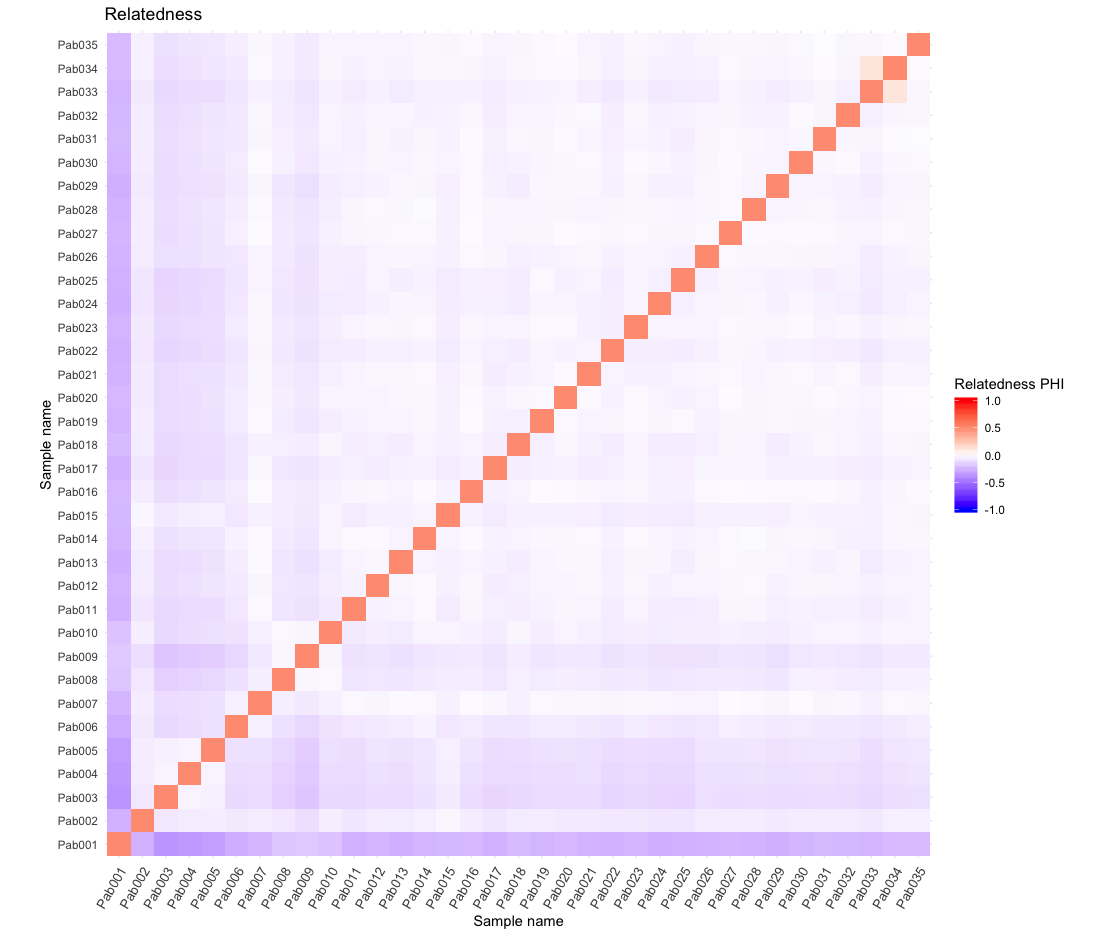


Figure S2. Estimated genetic relatedness between each pair of 35 individuals using 294 million SNPs.

Figure S3. Schematic diagram of tested demographic models used in fastsimcoal2. All models included three current-day populations that were derived from a common ancestral population that experienced an ancient population bottleneck. Following the bottleneck, the Central-Europe population was assumed to diverge from the ancestral population, followed by the divergence between of the Finland and Sweden-Norway populations. The models differed depending on population sizes following divergence and whether individual populations went through further bottlenecks. Framed model ‘Pop012-Bot’ was the best-fitting model (see Supplementary Table S3).

Figure S4. Scatterplots of intronic nucleotide diversity, intergenic nucleotide diversity versus gene density for Central-Europe population (dark red), Finland population (dark blue), and Sweden-Norway population (dark green) across the *P. abies* genome. The Spearman correlation coefficient (r) and linear regression (r^2^) are shown in the top right of each plot. Linear regression lines are depicted in grey.

Figure S5. Scatterplots of the population-scaled recombination rates versus gene density for Central-Europe population (dark red), Finland population (dark blue), and Sweden-Norway population (dark green) across the *P. abies* genome. The Spearman correlation coefficient (r) and linear regression (r^2^) are shown in the top right of each plot. Linear regression lines are depicted in grey.

Figure S6. Heatmap of spearman correlation tests between pairwise factors. Spearman correlation coefficient (r) is shown in each block of pairwise correlation test.
